# An analysis of research biopsy core variability from over 5000 prospectively collected core samples

**DOI:** 10.1038/s41698-021-00234-8

**Published:** 2021-10-27

**Authors:** Deepak Bhamidipati, Anuj Verma, Dawen Sui, Dipen Maru, Grace Mathew, Wenhua Lang, Juan Posadas, Joshua Hein, Scott Kopetz, Andrew Futreal, Ignacio I. Wistuba, Sanjay Gupta, J. Jack Lee, Michael J. Overman, Alda L. Tam

**Affiliations:** 1 Department of Internal Medicine, Baylor College of Medicine, Houston, TX USA; 2 Department of Translational Molecular Pathology, The University of Texas MD Anderson Cancer Center, Houston, TX USA; 3 Department of Biostatistics, The University of Texas MD Anderson Cancer Center, Houston, TX USA; 4 Department of Pathology, Anatomical, The University of Texas MD Anderson Cancer Center, Houston, TX USA; 5 Department of Quantitative Research Computing, The University of Texas MD Anderson Cancer Center, Houston, TX USA; 6 Department of Interventional Radiology, The University of Texas MD Anderson Cancer Center, Houston, TX USA; 7 Department of Gastrointestinal Medical Oncology, The University of Texas MD Anderson Cancer Center, Houston, TX USA; 8 Department of Genomic Medicine, The University of Texas MD Anderson Cancer Center, Houston, TX USA

**Keywords:** Molecular medicine, Translational research

## Abstract

Factors correlated with biopsy tissue adequacy and the prevalence of within-biopsy variability were evaluated. Totally, 1149 research biopsies were performed on 686 patients from which 5090 cores were assessed. Biopsy cores were reviewed for malignant percentage (estimated percentage of cells in the core that were malignant) and malignant area (estimated area occupied by malignant cells). Linear mixed models and generalized linear mixed models were used for the analysis. A total of 641 (55.8%) biopsies contained a core with <10% malignant percentage (inadequate core). The chance of an inadequate core was not influenced by core order, though the malignant area decreased with each consecutive core (*p* < 0.001). Younger age, bone biopsy location, appendiceal tumor pathology, and responding/stable disease prior to biopsy increased the odds of a biopsy containing zero adequate cores. Within-biopsy variability in core adequacy is prevalent and suggests the need for histological tumor quality assessment of each core in order to optimize translational analyses.

## Introduction

The molecular characterization of tumors has become a critical component of cancer care. Clinical practice guidelines for several solid tumors, including lung, breast, and colon cancer, incorporate routine molecular testing to inform treatment decisions^1–3^. The understanding of molecular and immune mechanisms of the tumor and its microenvironment are often important correlative endpoints for clinical trials^4^. Tumor tissue for these molecular assays is typically obtained through image-guided biopsies, a technique routinely used in the diagnosis and increasingly applied for research purposes^5,6^.

Biopsies obtained for research purposes require substantially more tumor tissue than what would be expected for a diagnostic biopsy due to the variety of molecular assays performed for research protocols. Moreover, decisions regarding optimal treatment for various malignancies rely on an increasing array of molecular tests, including immunohistochemistry (IHC), fluorescence in situ hybridization (FISH), and next-generation sequencing (NGS) for which high-quality samples are necessary. Each molecular test will have unique minimum tissue requirements depending on the methods and testing platform used; conventional DNA sequencing approaches such as Sanger necessitate greater than 15–25% tumor nuclei as compared to the much lower requirement of ~5% for contemporary NGS platforms^7–9^. Though molecular tests are becoming more efficient, higher tissue quantities generally enable improved accuracy^10,11^, and newer techniques, such as single-cell RNA sequencing, further propagate the need for high-quality biopsy samples^12,13^. Given the breadth of molecular tools available as well as constraints of current molecular analysis platforms, research biopsies are frequently found to be insufficient for analysis, with specimen inadequacy rates reaching up to one-third of all samples in some studies^14–17^. Limited tumor yield may be one of several factors contributing to the poor publication rates of analyses expected from research biopsies^18,19^.

Apart from needle gauge and lesion size, other factors associated with tumor yield remain largely undefined^20–24^. Moreover, the current workflow assumes relatively little core-to-core variability with respect to the presence of adequate tumor tissue, with the disposition of the biopsy cores being assigned a priori to a specific molecular test. This approach risks inaccurately estimating tumor tissue within a core which has implications for sample processing and ultimately, for the success of correlative endpoints. The goal of the present study is to identify the prevalence of within-biopsy variability and what role patient, tumor, and procedure characteristics have in determining biopsy tissue adequacy.

## Results

### Study population and biopsy safety

A total of 1149 biopsies were performed on 686 patients across 28 clinical studies, from which 5090 cores were analyzed. 1019 (88.7%) of these biopsies were performed by IR. Four or more cores were obtained for 982 biopsies (85.5%). A total of 488 (42.5%) were serial biopsies obtained after the baseline biopsy. Twenty-gauge, 18-gauge, 16-gauge, and 14-gauge needles were used in 160 (13.9%), 886 (77.1%), 9 (0.9%), and 94 (8.2%) biopsies respectively. Notably, over half of the biopsies of untreated lesions (62/117) were breast biopsies which were performed using larger 14-gauge needles. Other demographic and procedure characteristics are summarized in Table 1. In total there were 37 biopsy complications (3.2%) of which 3 (0.3%) were CTCAE grade 3 adverse events consisting of one bleeding complication requiring treatment with transfusion and two incidences of arrhythmia. There were no Grade 4 or Grade 5 adverse events. Complications were directly related to the location of the biopsy (Supplementary Table 2) and highest for intrathoracic biopsies at 13.2% (20/151 biopsies), followed by solid organ biopsies at 2.9% (11/374 biopsies), and deep abdomino-peritoneal biopsies at 1.9% (6/308). Pneumothorax and bleeding were the most common types of complications.

**Table 1:** Patient and biopsy description.

*Total number of patients*	686
Male gender (%)	308 (44.9%)
Median age (IQR)	60 (48–68)
Median BMI (IQR)	26 (23–31)
Median lines of chemotherapy (range)	1 (0–11)
*Total number of biopsies*	1149
Serial biopsy	488 (42.5%)
IR performed biopsy	1019 (88.7%)
Biopsy of primary tumor	167 (14.5%)
Received RT to lesion	34 (3.0%)
*Disease status at time of biopsy*^a^	
Untreated disease	117 (10.3%)
Partially responding	63 (5.5%)
Stable disease	189 (16.6%)
Progressive disease	773 (67.7%)
*IR biopsy by physician expertize*	
Experienced specialist (>10 years) and with trainee	178 (17.5%)
Experienced specialist (>10 years) and without trainee	482 (47.3%)
Experienced specialist (≦10 years) and with trainee	151 (14.8%)
Experienced specialist (≧10 years) and without trainee	208 (20.4%)
*Needle size*	
14-gauge	94 (8.2%)
16-gauge	9 (0.8%)
18-gauge	886 (77.1%)
20-gauge	160 (13.9%)
*Tumor pathology group*	
Anal cancer	60 (5.2%)
Appendiceal tumor	76 (6.6%)
Bone and soft tissue tumor	164 (14.3%)
Breast cancer	130 (11.3%)
Colorectal cancer	114 (9.9%)
Neuroendocrine tumor	89 (7.7%)
Ovarian cancer	50 (4.4%)
Pancreaticobiliary cancer	79 (6.9%)
Thyroid cancer	61 (5.3%)
Other^b^	326 (28.3%)
*Biopsy location*	
Bone	10 (0.9%)
Breast	93 (8.1%)
Chest-intrathoracic	151 (13.1%)
Abdominal-pelvic deep tissue	308 (26.8%)
Solid organ	374 (32.6%)
Superficial	194 (16.9%)
Thyroid	19 (1.7%)
Median lesion size in cm^2^ (IQR), (mean, SD)^c^	3.0 (2.0–4.2), (3.6, 2.7)
Average malignant area per biopsy in mm^2^ (IQR), (mean, SD)	2.7 (0.8–5.3), (3.4, 3.2)
Total number of cores analyzed	5090
Biopsies with inadequate core(s)	641 (55.8%)
*Number of cores per biopsy*	
1 core	67 (5.8%)
2 cores	35 (3.0%)
3 cores	65 (5.7%)
4 cores	232 (20.2%)
5 cores	703 (61.2%)
6 cores	28 (2.4%)
7 cores	3 (0.3%)
8 cores	4 (0.3%)
9 cores	0 (0%)
10 cores	12 (1%)

^a^Seven patients with missing information

^b^Other: head and neck SCC (*n* = 19, 1.7%), CNS malignancy (*n* = 2, 0.2%), leukemia and lymphoma (*n* = 2, 0.2%), endometrial cancer (*n* = 26, 2.3%), hepatocellular carcinoma (*n* = 27, 2.4%), renal cancer (*n* = 39, 3.4%), skin malignancy (*n* = 39, 3.4%), thoracic malignancy (*n* = 39, 3.4%), small bowel adenocarcinoma (*n* = 40, 3.5%), cervical cancer (*n* = 41, 3.6%), peritoneal malignancy (*n* = 42, 3.7%), gastroesophageal cancer (*n* = 5, 0.4%), and genitourinary cancer (*n* = 5, 0.4%)

^c^Seventy-eight patients with missing information.

### Biopsy core adequacy

Tumor content assessment for the first five cores from all biopsies is described in Supplementary Table 2. For all cores (*n* = 5090), the median core area was 6.0 mm^2^ (interquartile range, IQR: 3–10); median core area with a 14-gauge, 18-gauge, and 20-gauge needle was 9, 6, and 3 mm^2^ respectively. The median tumor area was 5.0 mm^2^ (IQR: 0.16–9.0) and the median malignant area was 2.25 mm^2^ (IQR: 0–5.4). The median percent of each core that was the tumor was 100% (IQR: 16.3–100) and the median malignant percent was 29% (IQR: 0.5–70).

**Fig. 1:**
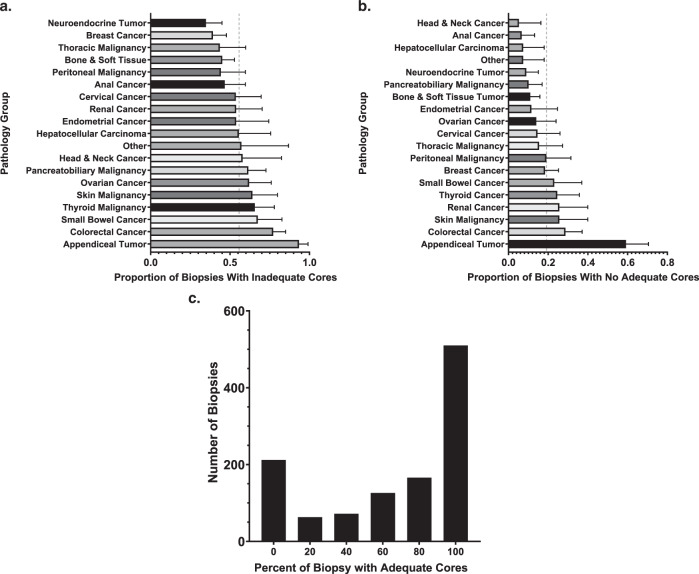
Proportion of inadequate cores per biopsy. **a** The proportion of biopsies for each pathology group containing at least one inadequate core is displayed. The dotted line references the proportion of all biopsies with at least one inadequate core. **b** The proportion of biopsies for each pathology group containing no adequate cores is displayed. The dotted line references the proportion of all biopsies with no adequate cores. **c** Displays the distribution of inadequate cores per biopsy. Each bin refers to the proportion of the biopsy cores in a single biopsy that contains adequate (>10%) malignant cells. For example, if 3 out of 5 biopsy cores (60%) contained >10% malignant cell, it would be counted in the bin “60–79%”.

Adequate cores were defined as cores containing >10% malignant percentage. Of the 1149 biopsies, all cores were adequate in 508 (44.2%), cores were a mixture of adequate and inadequate cores in 429 (37.3%), and all cores were inadequate in 212 (18.5%) biopsies. Within the subset of the biopsies in which all cores were inadequate, zero malignant cells were seen in 135 biopsies. Thus, in 937 (81.5%) biopsies at least one core was adequate and in 641 (55.8%) biopsies at least one core was inadequate. In total, 1801 (35.4%) cores were inadequate and not dependent on core order (*p* = 0.645) (Supplementary Table 3). Notably, over half (*n* = 49, 59.2%) of all appendiceal tumor biopsies did not contain any adequate cores while greater than 90% of anal, breast, head, and neck, hepatocellular, and neuroendocrine tumor biopsies obtained at least one adequate core. The proportion of biopsies containing inadequate cores or adequate cores by the pathology group and the distribution of the proportion of adequate cores per biopsy are displayed in Fig. 1.

### Effects of core position on tumor content assessments

**Fig. 2:**
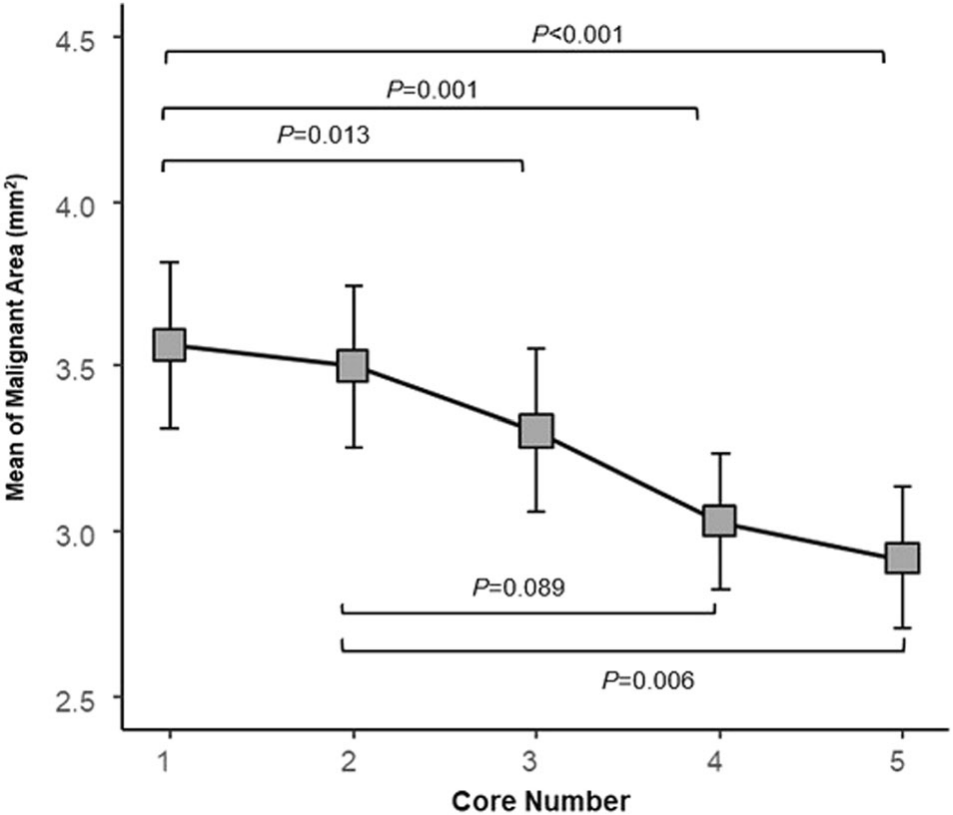
Comparison of malignant area content by core number. The figure shows the mean of the malignant area with SD. *p* Values are from Tukey adjusted methods controlling for covariances.

The malignant percentage did not vary according to the order in which cores were obtained (*p* = 0.821) nor did the rate of inadequate cores (*p* = 0.645). Interestingly, we did note that both core area and tumor area decreased with increasing core number (*p* < 0.001 and *p* < 0.001). When evaluating the amount of malignant area present on each core, decreasing amounts of the malignant area were seen with increasing core number (Fig. 2 and Supplementary Table 4): the third, fourth, and fifth cores contained statistically significant lower malignant area than the first core (*p* = 0.013, *p* = 0.001, *p* < 0.001, respectively for each comparison). Interestingly, the malignant percentage did not vary according to the order in which cores were obtained (*p* = 0.821) nor did the rate of inadequate cores (*p* = 0.645).

### Assessment of factors contributing to the malignant tumor content of core biopsies

**Fig. 3:**
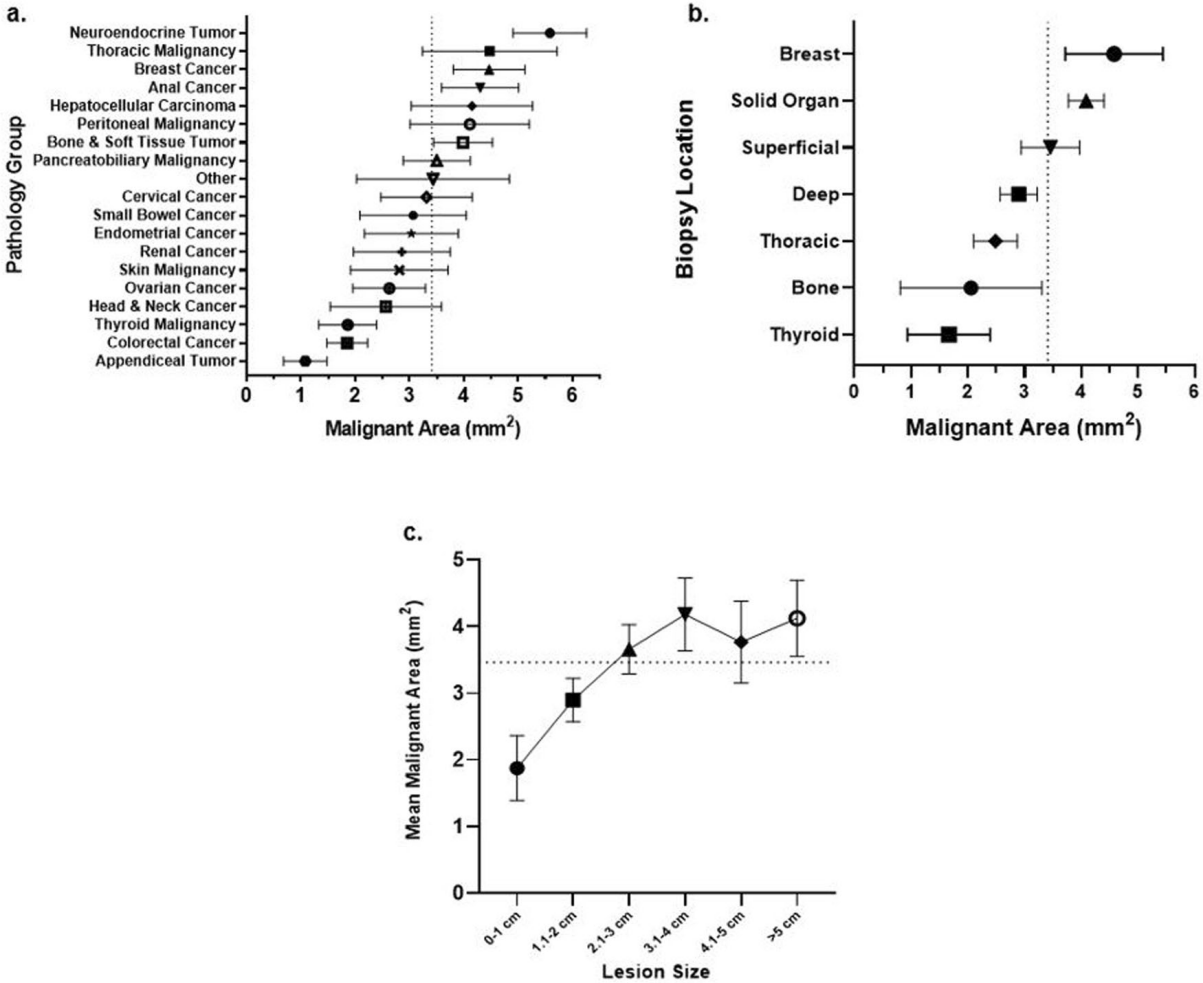
Malignant area by tumor pathology, biopsy location, and lesion size. **a** The average malignant area per core for each biopsy stratified by pathology group is displayed above along with 95% confidence interval estimates. The dotted line represents the average malignant area per core for all biopsies. **b** The average malignant area per core for each biopsy stratified by location is displayed above along with 95% confidence interval estimates. The dotted line represents the average malignant area per core for all biopsies. Note: The majority of breast biopsies were performed using 14-gauge needles and the majority of thyroid and thoracic biopsies were performed using 20-gauge needles. **c** The average malignant area per core for each biopsy stratified by lesion size is displayed above along with 95% confidence interval estimates. The dotted line represents the average malignant area per core for all biopsies where lesion size was available.

All multivariate analyses were performed using data from only IR biopsies (*n* = 1019). To evaluate factors correlating with the number of malignant cells obtained from research biopsies, we used the surrogate of malignant area (the estimated area of each core occupied by malignant cells). Using a linear mixed model, the factors influencing average malignant area on multivariable analysis were age (*p* < 0.001), body mass index (BMI) (*p* = 0.021), needle gauge (*p* = 0.034), tumor pathology (<0.001), lesion size (<0.001), biopsy site (*p* = 0.005), and biopsy time point (*p* = 0.027) (Supplementary Table 5). Malignant area decreased with older age, higher BMI, smaller needle gauge, and post-treatment biopsy timepoint. Bone biopsy location and appendiceal pathology were associated with the lowest malignant area. Malignant area correlations with pathology type, biopsy location, and lesion size are shown in Fig. 3.

On multivariable analyses, age (*p* < 0.001), lesion size (*p* = 0.006), and tumor pathology (*p* < 0.001) influenced the odds of a biopsy containing at least one inadequate core (Supplementary Table 6); younger age, smaller lesion size, and appendiceal/colorectal pathology increased odds of a biopsy containing at least one inadequate core. Age (*p* = 0.037), biopsy location (*p* = 0.04), tumor pathology (*p* < 0.001), and disease status (*p* = 0.046) influenced odds that a biopsy contained zero adequate cores (Supplementary Table 7); younger age, biopsy of bone, appendiceal/colorectal pathology, and stable/responding disease increased odds of a biopsy containing zero adequate cores. The proportion of biopsies containing inadequate cores by the pathology group is displayed in Fig. 1.

## Discussion

Though research biopsies are increasingly incorporated into clinical trials, the lack of research biopsy result reporting is a major impediment toward gaining the generalizable knowledge that underlies the ethical framework to conduct research biopsies on patients^18^. Omission of pharmacodynamic data in clinical trial publications is prevalent, with authors often citing poor or incomplete analyses as an explanation for this lack of reporting^25^. In this analysis, we sought to understand the pre-analytical factors related to the amount of tumor present within research biopsy cores. Where adequacy was defined by the tumor cellularity requirements for successful NGS, this analysis found that an inadequate core (<10% malignant percentage), was present in 55.8% of biopsies and that 18.5% of biopsies contained all inadequate cores. Interestingly, the chance of an inadequate core did not vary by core position, but the malignant area did decrease with successive cores as overall core size decreased. This high rate of intra-biopsy core variability questions the common practice of assessing a biopsy’s success based on a single core, the a priori assignment of cores to molecular tests solely by core order, and suggests the need for tumor quality assessment for individual cores from research biopsies. Such assessment would enable optimal matching of core tumor tissue to analytical test tumor requirements.

Though the finding that the risk of an inadequate core did not vary by core position is surprising, the finding that the malignant area decreases with subsequent cores is expected. Typically, the first core is obtained from an untouched tumor but results in manipulation of the tumor and surrounding environment in the process. Subsequent cores are thus at a progressively increased risk of passing through areas devoid of previously obtained tumor tissue in addition to debris generated from the previous biopsy passes. Awareness that successive passes of the biopsy needle should be expected to yield diminishing returns could lead to intra-procedural technique modifications, such as tilting the guide needle to sample different areas of the tumor or adjusting the cutting edge of the core biopsy needle to sample from untouched areas, that may mitigate the loss of malignant area. This finding would also suggest that, in the absence of individual core quality assessment, the initial cores obtained during a research biopsy should be allocated to the molecular assays that require the highest tumor tissue quantities.

Approximately, 37.3% of biopsies were found to contain both adequate and inadequate cores, and there was no correlation between core position and risk of inadequacy. The unpredictability of core adequacy highlights the need for the integration of pathology quality assessment as a best practice to optimize the usage of tissue samples collected for clinical trials. Quality assessment of both the FFPE and frozen cores as performed in this study can permit for appropriate matching of individual core biopsies to specific molecular assays to improve testing success across the entire specimen. While some clinical trials have incorporated the use of rapid-on-site cytological evaluation, this test confirms malignancy but usually only from one sample and does not address the intra-core variability concerns demonstrated by this data. Eventually, novel techniques such as real-time fluorescence confocal digital microscopy may permit point-of-care testing to determine core biopsy adequacy at the time of tissue acquisition^26^.

Authors have identified the absence of high-quality biospecimens as a significant barrier to the development and validation of biomarkers^27,28^ and our finding that 18.5% of biopsies contained all inadequate cores underscores the need for a better understanding of pre-analytical factors contributing to biopsy yield. The multivariate analyses on factors associated with all inadequate cores within a biopsy sample and those associated with decreasing malignant area overlap and can be discussed in the context of three broad categories: tumor pathology, factors contributing to the technical success of the biopsy procedure, and patient factors.

Biopsy adequacy varied significantly by tumor pathology, as indicated by the remarkably high yields seen with neuroendocrine tumors in contrast to appendiceal tumors. The difference in the tumor microenvironments is a driver for biopsy adequacy: neuroendocrine tumors tend to be well-circumscribed, with very little intervening desmoplastic stroma which differs from appendiceal neoplasms, which are often characterized by sparse malignant cells surrounded by large acellular areas of mucin^29,30^.

Lesion size, biopsy location, needle size, and patient BMI are all factors that can contribute to the technical success of a biopsy procedure. Larger lesions provide better targets and can accommodate larger needle sizes which in turn yield greater amounts of tissue. Similar to the findings of Kim et al. and Li et al.^22,24^, biopsies of subcentimeter lesions in this study returned substantially less tissue than biopsies obtained from larger lesions, with the mean malignant area from biopsy cores from lesions 0 to 1 cm is approximately half of what was obtained from biopsy cores from lesions 2.1–3 cm. In contrast to bony lesions, obtaining multiple cores from tumors in solid organs and other soft tissue is easier and no decalcification step, which may contribute to the degradation of DNA and protein, is required in sample processing. Patients with lower BMI present less of a technical challenge both in terms of visualization of lesions using imaging guidance as well targeting of the lesions for sampling; the need to traverse less tissue to a well-defined target may decrease the likelihood of off-target sampling.

Patient factors such as age and disease response status at the time of biopsy are also contributing factors. The associations between younger age and zero biopsy adequacy and older age and higher malignant area are less obvious. Perhaps this may be partly attributed to age-related physical changes, such as decreased skin elasticity and muscle mass resulting in a lower BMI and facilitating needle access to the lesion. Alternatively, this could relate to selection bias, with younger patients enrolled into clinical trials despite suboptimal lesions for biopsy. Patients in a stable or responding phase of their disease may have a fewer number of lesions to select from for biopsy and potentially less viable tissue, thus contributing to the risk of having a research biopsy with zero adequate cores. In contrast, patients undergoing a baseline biopsy are usually doing so to start enrollment on a trial, presumably because they have progressed through their last line of therapy and therefore, may have more disease sites amenable to biopsy in addition to proven viability as evidence by radiographic growth of the lesions.

There were several strengths of our study, namely the large, diverse sample size allowing for a thorough comparison between different pathologies and demographic characteristics. Most biopsies were also performed by the same department, using standardized protocols, decreasing the risk of bias. Our findings demonstrate the safe use of 18-gauge biopsies (77.1% of biopsies) to obtain at least 5 cores (65.2% of biopsies) as the major complication rate was 0.3% with no CTCAE events > Grade 4. These findings compare favorably to the expected rates for diagnostic biopsy^31^ and the recently reported molecular triage study (MOSCATO-01)^32^.

The major limitation was the inability to directly measure the clinical impact of the variable tissue yields such as the ability to successfully perform NGS or other molecular tests; once the biopsy tissue was reviewed for adequacy, samples were sent for protocol-specific testing which was not standardized across the clinical trials. Due to the various protocol-specific uses of tumor tissue for translational analyses, a standardized threshold will always be imprecise as the exact threshold for adequacy will relate both to the testing platform and minimum tissue requirements. As an example, two of the 28 clinical trials have published results related to translational endpoints and these two trials demonstrate the variability of biopsy analysis related to both tumor type and testing methodology. In one clinical trial conducted in colorectal cancer 10 of 21 pre-treatment biopsies of liver metastases were eligible for RNA sequencing with reasons related to the normal liver in 4, necrotic tumor only in 2, and minimal tumor amount in 5^33^. However, in a second clinical trial conducted in primary breast cancer, 98 of 105 pre-treatment biopsies were able to undergo TCR clonality analysis^34^. In addition, the malignant area was used as a surrogate for the number of malignant cells, which for certain analyses would be a more optimal measure of the tumor amount present. Finally, we recognize that for certain analyses that require viable cells, such as flow cytometry or organoids, it is not possible to generate an hematoxylin and eosin (H&E) to enable core tumor assessment.

The National Cancer Institute endorses the value of the multidisciplinary biopsy team with the understanding that standard operating procedures for tissue acquisition and processing can achieve better research biopsy results^14,35^. The role of the interventional radiologist for optimizing lesion selection and mitigating patient risk has been previously defined^36^. This study presents the value proposition of robust pathology collaboration. In demonstrating high intra-biopsy variability, with greater than half of all biopsies containing one or more inadequate cores, this study highlights a potential strategy to maximize the utility of research biopsies: the implementation of routine quality assessment of research biopsy cores with the appropriate matching of individual core biopsies to specific molecular assays to improve testing success across the entire specimen. These data summarized in the manuscript also highlight that the research expectations of many clinical trials may be too ambitious and not realistic with respect to the number of proposed tests given the prevalence of inadequate specimens.

## Methods

### Patients

This was HIPPA compliant and was approved by the University of Texas MD Anderson Cancer Center Institutional Review Board. Written informed consent was waived for this retrospective analysis. In this study, a prospective research biopsy database at the University of Texas MD Anderson Cancer Center (Houston, TX) was used to identify research biopsies obtained for clinical trials affiliated with the Adaptive Patient-Oriented Longitudinal Learning and Optimization (APOLLO) program between August 2016 and August 2019. Consent was obtained from all human participants enrolled in clinical trials affiliated with the APOLLO program for the use of their tissue for future research purposes. Patients included in the database were enrolled in 27 phase I and II studies (Supplementary Table 1) and one clinical trial companion biopsy collection trial. For each trial, enrolled patients typically underwent a pre-treatment biopsy with the possibility of additional biopsies at multiple time points either while on treatment and/or at the time of progression. Clinical and procedure characteristics were obtained for each biopsy procedure from a review of the electronic medical record.

### Biopsies

Non–image-guided biopsies were excluded from this analysis. Breast biopsies (*n* = 93) were performed under US guidance by breast radiology while thyroid (*n* = 19) and some superficial biopsies (*n* = 18) were performed by neuroradiology. Most biopsies (*n* = 1019) were performed by the Interventional Radiology (IR) department under CT- or US guidance using previously described techniques, including the assignation of a lesion score to denote whether the lesion was expected to yield sufficient material for biomarker analysis^36,37^. All IR biopsies were performed using a co-axial technique, which allows for the acquisition of multiple biopsy samples. Co-axial guide needles were either 19-gauge for lung biopsies in which 20 G cores were obtained or 17-gauge for biopsies in which 18 G cores were obtained. The core needle would be moved slightly to optimize tissue collection, but the co-axial needle was not moved to sample from multiple foci within the tumor. Typically, a 20-gauge core biopsy needle was used to acquire samples from intra-thoracic lesions and an 18-gauge core biopsy needle was used for all other lesions. For each IR biopsy, five or more cores were obtained when feasible and numbered based on the order of collection. Similar procedural processes were used for breast and thyroid biopsies, however, 14-gauge needles were routinely used for breast biopsies and 20-gauge for thyroid.

### Pathology review

**Fig. 4:**
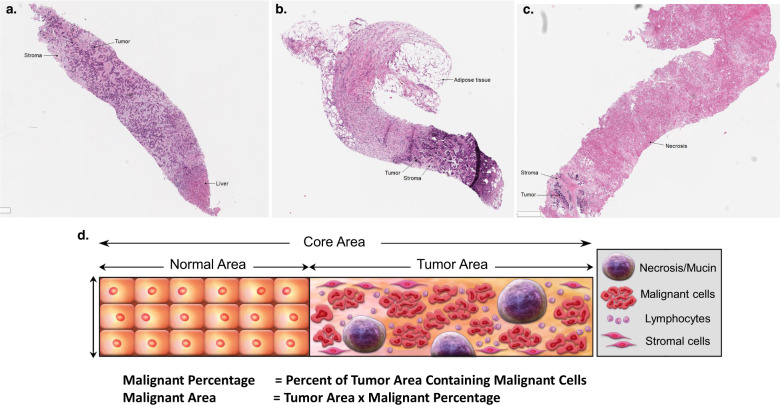
Biopsy core. **a** A photomicrograph of a representative core biopsy containing 70% malignant cells. **b** A photomicrograph of a representative core biopsy containing 30% malignant cells. **c** A photomicrograph of a representative core biopsy containing 5% malignant cells. **d** The illustration demonstrates the various cellular elements that can be included in a biopsy core specimen.

Fresh research biopsy cores were submitted to pathology for processing and quality assessment review. Typically, two cores were formalin-fixed and paraffin-embedded (FFPE) onto a tissue cassette while the remaining cores were flash frozen. A representative illustration of tissue within a core biopsy, including cellular tumor, non-cellular tumor, and non-tumor components, is shown in Fig. 4. Cores sent for flow cytometry analysis were not processed for tumor content assessment. The following protocol was implemented for H&E preparation of frozen specimens: the tissue was retrieved from the cryovial and placed in OCT on a cryomold. The cryomold was placed on dry ice to solidify the OCT. The cryomold was then positioned in the center of a chuck which was then mounted in the microtome of the cryostat. Five-micron thick sections were cut and collected on a glass slide. The slide was then loaded on the Shandon Veristain Gemini Automatic stainer. The slide was first dipped in 95% alcohol and then washed in running water followed by a dip in hematoxylin. The slide was then washed again and dipped in clarifier reagent. The slide was washed again and dipped in bluing reagent and then washed again. It was then dipped in aqueous eosin followed by 3 dips in increasing concentration of 95–100% alcohol and 3 dips in xylene. The slide was then coverslipped and dried.

A trained pathologist (A.V.) performed a quality assessment of each core after H&E staining of a representative slide. Adequate cores were considered to contain >10% malignant percentage versus inadequate cores (<10% malignant percentage). In all, 10% malignant tumor was selected as a threshold as it has been shown to be sufficient for NGS using contemporary platforms, though a requirement of greater than or equal to 20% malignant tumor nuclei is suggested for some platforms^9,38^. The threshold was not based on requirements of other molecular tests such as IHC and FISH which generally require lower tumor cellularity. After review by a pathologist, biopsies were sent for protocol-specific testing.

### Statistical analysis

The associations between the factors of interest and continuous dependent outcomes were detected using linear mixed models (LMM) and the associations between the factors of interest and dichotomized dependent outcomes were detected using generalized LMM. The residual (restricted) maximum likelihood or residual (restricted) pseudo-likelihood estimation methods and KENWARDROGER^39^ degrees-of-freedom correction were also selected for modeling. Square root transformation for the continuous dependent outcomes was performed to make the residual of the model normal. Multicovariate analysis was then conducted using backward elimination based on the likelihood ratio test and including all the factors with *p*-value < 0.10 in the univariate analyses. Tumor pathology and histology were almost perfectly correlated (*p* < 0.0001), thus the univariate and multicovariate analyses were conducted for pathology alone. All analyses of modeling were adjusted for the time of biopsy regardless of significance status. Tukey adjustments for multiple testing were also made. A *p*-value of less than 0.05 was considered statistically significant. All statistical analyses were performed using SAS 9.4 for Windows (Copyright © 2011 by SAS Institute Inc., Cary, NC).

Only the quantities contained in the first five cores of each biopsy were compared due to the limited sample size after the fifth core. For multivariable analysis of factors related to biopsy tissue quantities, only IR biopsies (*n* = 1019) were analyzed; this decision was made to minimize potential confounders such as different protocols, techniques, and needle sizes (14-gauge for breast and 20-gauge for thyroid) implemented by non-IR departments.

To facilitate analysis, similar tumor types and biopsy sites were grouped into more inclusive categories. The following pathology groupings were used: genitourinary (prostate cancer and bladder cancer); bone and soft tissue (sarcoma [multiple types], chondroma); gastroesophageal (esophageal adenocarcinoma, gastric cancer); CNS (glioblastoma, ependymoma); pancreaticobiliary cancer (pancreatic adenocarcinoma, cholangiocarcinoma); peritoneal malignancy (peritoneal malignancy mesothelioma, peritoneal malignancy serous adenocarcinoma); thoracic (non-small cell lung cancer, pleural mesothelioma); skin (melanoma, Merkel cell carcinoma); leukemia and lymphoma (diffuse large b-cell lymphoma, acute lymphoblastic leukemia); renal (renal cell carcinoma, renal medullary carcinoma); thyroid (anaplastic thyroid cancer, papillary thyroid cancer, medullary thyroid cancer). Other categories not specified contained a single pathologic variant. The following location groupings were used: chest-intrathoracic (lung, intrathoracic lymph node, pleura, pericardium); abdominopelvic deep tissue (peritoneal malignancy, retroperitoneal malignancy, extraperitoneal malignancy); solid organ (liver, spleen, kidney, pancreas, adrenal gland, stomach); superficial (superficial lymph node, chest/abdominal wall, joint/extremity). bone, breast, and thyroid biopsies contained no subgroups.

For analyses, pathology groups containing less than ten patients (CNS, genitourinary, hematologic, and gastroesophageal malignancies) were grouped as “Other” due to the small sample size. IR biopsies performed using 14-gauge and 16-gauge needles were also combined were indicated for some of the analyses due to similar considerations.

### Reporting summary

Further information on research design is available in the Nature Research Reporting Summary linked to this article.

## Supplementary information

Reporting Summary

Supplementary Information

## Data Availability

The data set will be made available to qualified medical researchers by request to the corresponding author’s Tam or Overman, though patient-level data linkage to specific clinical trials cannot be provided.
