# Supplementary material for: An analysis of research biopsy core variability from over 5000 prospectively collected core samples
Source: NPJ Precis Oncol. 2021 Oct 27;5:94. doi: 10.1038/s41698-021-00234-8 (PMC8551285; doi:10.1038/s41698-021-00234-8)
Supplement: Supplementary file 2 — Supplementary Information [file 41698_2021_234_MOESM2_ESM.pdf]

Data Supplement

An Analysis of Research Biopsy Core Variability From Over 5000 Prospectively Collected Core Samples

Table of Contents:

**Supplementary Table 1.** Clinical Trials Patients Enrolled In	Page 2
**Supplementary Table 2.** Biopsy Complications Stratified by Location	Page 4
**Supplementary Table 3.** Summary Data by Core Number	Page 5
**Supplementary Table 4.** Pairwise Comparisons of Cores	Page 6
**Supplementary Table 5.** Univariate and Multivariable Analysis of Factors Related to square-root transform Malignant Area for IR Biopsies	Page 7
**Supplementary Table 6.** Univariate and Multivariable Analysis of Factors Related to Presence of Inadequate Core(s) for IR Biopsies	Page 11
**Supplementary Table 7.** Univariate and Multivariable Analysis of Factors Related to Zero Adequate Core(s) for IR Biopsies	Page 14

Supplementary Table 1. Clinical Trials Patients Enrolled In

NCT Number	Start Year	Trial Title
NCT02276443	2014	ARTEMIS: A Robust TNBC Evaluation fraMework to Improve Survival.
N/A	2014	Longitudinal Biospecimen Acquisition for All Tumor Types
NCT02754856	2015	Pilot Study Assessing the Safety and Tolerability of the Neoadjuvant Use of Tremelimumab (anti-CTLA-4) plus Durvalumab (MEDI4736) (anti-PD-L1) in the Treatment of Resectable Colorectal Cancer Liver Metastases.
NCT02815995	2015	A Phase II Multi Arm Study to Test the Efficacy of Immunotherapeutic Agents in Multiple Sarcoma Subtypes
NCT02940496	2016	A Phase II Study of Pembrolizumab (MK-3475) in Hepatitis C Virus Positive and Negative Subjects with Advanced Hepatocellular Cancer Carcinoma Who Progressed on or Were Intolerant to First-Line Systemic Therapy
NCT02907099	2016	A Phase IIb Pilot Study to Assess the Efficacy, Safety and Pharmacodynamics Effects of Pembrolizumab and BL-8040 in Patients with Metastatic Pancreatic Cancer
NCT02989064	2016	Phase 1 Cell Dose Escalation Study to Assess the Safety and Tolerability of Genetically Engineered Mage-A10 C796t in Hla-A2+ Subjects with Mage-A10 Positive Urothelial, Melanoma or Head and Neck Tumors
NCT03074513	2016	A Phase II, Single-Arm Open-Label Study Of The Combination Of Atezolizumab And Bevacizumab In Rare Solid Tumors
NCT03175432	2016	Phase II Study of BEvacizumab (Avastin) in Combination with ATezolizumab or Atezolizumab (Tencentriq) and Cobimetinib (Cotellic) in Patients with Untreated Melanoma Brain Metastases (TACo-BEAT-MBM).
NCT03174197	2016	Phase I/II Study to Evaluate the Safety and Clinical Efficacy of Atezolizumab (aPDL1) in Combination with Temozolomide and Radiation in Patients with Newly Diagnosed GBM
NCT03108131	2016	A Phase II, Open-label, Single-arm, Multi-cohort, Proof-of-principle Study to Investigate the Efficacy of Cobimetinib and Atezolizumab in Advanced Rare Tumors
NCT03202316	2016	A phase II study of triple combination of Atezolizumab cobimetinib + eribulin (ACE) in patients with recurrent/metastatic inflammatory breast cancer
NCT03395847	2016	A Pilot Study of Pembrolizumab Monotherapy in Patients with Previously Treated Advanced Gastroesophageal Adenocarcinoma
NCT02992743	2016	A Pilot Study of NY-ESO-1c259T Cells in Subjects with Advanced Myxoid/ Round Cell Liposarcoma
NCT03181100	2016	Atezolizumab Combinations with Chemotherapy for Anaplastic and Poorly Differentiated Thyroid Carcinomas
NCT03132922	2016	Phase 1 Dose Escalation, Multi-tumor Study to Assess the Safety, Tolerability and Antitumor Activity of Genetically Engineered MAGE-A4c1032T in HLA-A2+ Subjects with MAGE-A4 Positive Tumors
NCT03217747	2017	Phase I/II Study to Evaluate the Safety and Tolerability of Avelumab in Combination with Other Anti-Cancer Therapies in Patients with Advanced Malignancies
NCT03307616	2017	Phase II Study of Neoadjuvant Checkpoint Blockade in Patients with Surgically Resectable Undifferentiated Pleomorphic Sarcoma and Dedifferentiated Liposarcoma
NCT03274258	2017	Phase II Trial of Nivolumab plus Ipilimumab in Patients with Renal Medullary Carcinoma
NCT03436563	2017	A Phase Ib/II Trial of M7824 in Solid Tumors with Microsatellite Instability with Consensus Molecular Subtype 4 Metastatic Colorectal Cancer in Combination with Radiation, or in Colorectal Cancer Patients with Detectible Circulating tumor DNA following Definitive Therapy.

NCT03524170	2017	RACHEL1: A Phase I Radiation and CHEckpoint bLockade trial in patients with metastatic hormone receptor positive, HER2 negative breast cancer
NCT03579472	2017	A Phase Ib trial of M7824 and Eribulin in Patients with Metastatic Triple Negative Breast Cancer (TNBC)
NCT03620201	2017	A Pilot Single Arm Open Label Trial Evaluating M7824 (Anti-PD-L1/TGF-Beta TRAP) in a Window Setting in Patients with Stage II-III HER2/neu Positive (HER2+) Breast Cancer (BC)
NCT03428126	2017	Phase II Study of Durvalumab (MEDI4736) (anti-PD-L1) and Trametinib (MEKi) in MSS Metastatic Colon Cancer
NCT03598595	2018	A Phase 1/2 Study of Gemcitabine and Docetaxel in Combination with Hydroxychloroquine (Autophagy Inhibitor) in Patients with Recurrent Osteosarcoma
NCT03637803	2018	A Phase I/II Open-Label, Safety And Preliminary Efficacy Study Of MRx0518 In Combination With Pembrolizumab In Patients With Advanced Malignancies Who Have Progressed On PD-1/PD-L1 Inhibitors
NCT03680521	2018	A Phase 2 Study of Sitravatinib in Combination with Nivolumab in Patients Undergoing Nephrectomy for Locally-Advanced Clear Cell Renal Cell Carcinoma
NCT03599765	2018	EXTernal Beam Radiation to Eliminate Nominal Metastatic Disease (EXTEND): A Randomized Phase II Basket Trial Assessing the Efficacy of Upfront Local Consolidative Therapy (LCT) for Oligometastatic Disease

Supplementary Table 2. Biopsy Complications Stratified by Location

Location	Number of Biopsies (%)	Minor Complications (CTCAE grade 1 or 2) (%)	Major Complications (CTCAE Grade 3) (%)

**Bone**	10 (0.9%)	0	0
**Breast**	93 (8.1%)	0	0
**Chest-Intrathoracic**	151 (13.1%)	20 (1.7%)	0
Lung	113	18	0
Intrathoracic Lymph Node	4	1	0
Pleura	33	1	0
Pericardial	1	0	0
**Abdomino-Pelvic Deep Tissue**	308 (26.8%)	5 (0.4%)	1 (0.09%)
Peritoneal Malignancy	154	2	1
RetroPeritoneal Malignancy	136	2	0
ExtraPeritoneal Malignancy	18	1	0
**Solid Organ**	374 (32.6%)	9 (0.8%)	2 (0.2%)
Liver	337	7	2
Spleen	1	0	0
Kidney	23	1	0
Pancreas	1	1	0
Adrenal Gland	9	0	0
Stomach	3	0	0
**Superficial**	194 (16.9%)	0	0
Superficial Lymph Node	71	0	0
Chest/Abdomen Wall	83	0	0
Joint/Extremity	40	0	0
**Thyroid**	19 (1.7%)	0	0
**Total**	1149 (100%)	34 (3%)	3 (0.3%)

Supplementary Table 3. Summary Data by Core Number

	All Cores (n=5090)	Core 1 (n=1061)	Core 2 (n=1078)	Core 3 (n=894)	Core 4 (n=1006)	Core 5 (n=907)
**Median Malignant Area in mm^2 (Mean)**	2.25 (3.40)	2.40 (3.78)	2.40 (3.65)	2.10 (3.36)	2.1 (3.12)	1.8 (2.98)
**Median Tumor Area in mm^2 (Mean)**	5.00 (5.53)	5.00 (6.01)	5.00 (5.92)	5.00 (5.47)	5.00 (5.13)	5.00 (5.05)
**Median Core Tissue Area in mm^2 (Mean)**	6.00 (6.16)	6.00 (7.18)	6.00 (7.03)	6.00 (6.47)	6.000 (6.19)	6.000 (6.01)
**Median Tumor Percentage (Mean)**	100 (73.1)	100 (73.7)	100 (73.4)	100 (72.9)	100 (72.5)	100 (74.0)
**Median Malignant Percentage (Mean)**	29.0 (36.1)	26 (35.8)	29 (35.8)	26.5 (36.3)	30 (36.7)	30 (36.1)
**Number of Inadequate Cores (Percentage)**	1801 (35.4%)	373 (35.1%)	368 (34.1%)	324 (36.2%)	356 (35.4%)	322 (35.5%)

Supplementary Table 4. Pairwise Comparisons of Cores

Table 5a. Pairwise Comparisons of Least Square Means for square-root transform of Malignant Area

		Univariate Result		Result adjusting covariances	
**Core #**	**Compared core #**	**Estimate(std)**	**TK adjusted P Value**	**Estimate(std)**	**TK adjusted P Value**
1	2	0.02 (0.04)	0.975	0.05 (0.04)	0.688
1	3	0.11 (0.04)	0.022	0.12 (0.04)	0.013
1	4	0.13 (0.04)	0.002	0.14 (0.04)	0.001
1	5	0.16 (0.04)	<0.001	0.18 (0.04)	<0.001
2	3	0.09 (0.04)	0.106	0.07 (0.04)	0.299
2	4	0.11 (0.04)	0.014	0.09 (0.04)	0.089
2	5	0.14 (0.04)	0.002	0.13 (0.04)	0.006
3	4	0.02 (0.04)	0.975	0.02 (0.04)	0.991
3	5	0.05 (0.04)	0.732	0.05 (0.04)	0.650
4	5	0.02 (0.04)	0.962	0.04 (0.04)	0.873
**Core #**	**Compared core #**	**Estimate(std)**	**TK adjusted P Value**	**Estimate(std)**	**TK adjusted P Value**
1	2	0.09 (0.12)	0.960	0.18 (0.13)	0.658
1	3	0.39 (0.13)	0.024	0.45 (0.14)	0.009
1	4	0.55 (0.13)	0.000	0.60 (0.13)	0.000
1	5	0.65 (0.13)	0.000	0.73 (0.13)	0.000
2	3	0.31 (0.13)	0.135	0.27 (0.14)	0.264
2	4	0.47 (0.13)	0.002	0.42 (0.13)	0.011
2	5	0.56 (0.13)	0.000	0.56 (0.13)	0.000
3	4	0.16 (0.13)	0.734	0.15 (0.14)	0.812
3	5	0.26 (0.14)	0.316	0.28 (0.14)	0.268
4	5	0.10 (0.13)	0.948	0.13 (0.14)	0.865

Supplementary Table 5. Univariate and Multivariable Analysis of Factors Related to square-root transform Malignant Area for IR Biopsies

Table 5a. Univariate Analysis of square-root transform Malignant Area

			95% CI			
**Factor**	**Levels**	**Estimate(Std)**	**Lower**	**Upper**	**Individual p Value**	**Overall p Value**
Gender	Male	0.00 (0.07)	-0.14	0.14	0.997	0.997
	Female	Ref	.	.	.	.
Biopsy of Primary	Yes	0.26 (0.14)	-0.01	0.52	0.058	0.058
	No	Ref	.	.	.	.
Previous RT	Yes	-0.02 (0.19)	-0.39	0.34	0.904	0.904
	No	Ref	.	.	.	.
Disease Status	SD	0.04 (0.08)	-0.12	0.19	0.645	0.161
	PR	-0.20 (0.13)	-0.46	0.06	0.139	.
	New Disease	0.19 (0.14)	-0.08	0.46	0.175	.
	PD	Ref	.	.	.	.
Tumor Pathology	Anal Cancer	0.01 (0.21)	-0.41	0.43	0.966	<0.001
	Appendiceal Tumor	-1.17 (0.20)	-1.57	-0.77	<0.001	.
	Bone & Soft Tissue	-0.19 (0.18)	-0.55	0.17	0.296	.
	Cervical Cancer	-0.25 (0.25)	-0.74	0.24	0.313	.
	Colorectal Cancer	-0.83 (0.19)	-1.20	-0.46	<0.001	.
	Endometrial Cancer	-0.25 (0.27)	-0.79	0.29	0.370	.
	Head & Neck Cancer	-0.43 (0.29)	-0.99	0.13	0.135	.
	Hepatocellular Cancer	-0.16 (0.26)	-0.67	0.34	0.522	.
	Neuroendocrine Tumor	0.31 (0.20)	-0.08	0.70	0.122	.
	Ovarian Cancer	-0.46 (0.23)	-0.91	-0.01	0.046	.
	Pancreaticobiliary Cancer	-0.25 (0.20)	-0.64	0.14	0.212	.
	Peritoneal Malignancy	-0.18 (0.23)	-0.63	0.26	0.418	.
	Renal Cancer	-0.57 (0.23)	-1.02	-0.12	0.014	.
	Skin Malignancy	-0.53 (0.23)	-0.98	-0.08	0.022	.
	Small Bowel Cancer	-0.48 (0.23)	-0.93	-0.03	0.038	.
	Thoracic Malignancy	-0.12 (0.23)	-0.57	0.33	0.605	.
	Thyroid Malignancy	-0.64 (0.25)	-1.13	-0.15	0.011	.
	Others	-0.32 (0.30)	-0.92	0.27	0.283	.
	Breast Cancer	Ref	.	.	.	.

			95% CI			
**Factor**	**Levels**	**Estimate(Std)**	**Lowe r**	**Uppe r**	**Individual p Value**	**Overall p Value**
Biopsy Location	Bone	-0.54 (0.31)	-1.15	0.06	0.079	<0.001
	Deep	-0.32 (0.08)	-0.48	-0.16	<0.001	.
	Superficial	-0.18 (0.09)	-0.37	0.00	0.053	.
	Thoracic	-0.35 (0.10)	-0.56	-0.14	0.001	.
	Thyroid	-1.73 (0.93)	-3.55	0.09	0.063	.
	Solid Organ	Ref	.	.	.	.
Experienced Proceduralist	Yes	-0.04 (0.05)	-0.14	0.07	0.513	0.513
	No	Ref	.	.	.	.
Presence of Assistant	Yes	-0.02 (0.06)	-0.13	0.10	0.759	0.759
	No	Ref	.	.	.	.
Needle Gauge	Size 14/16	0.51 (0.31)	-0.09	1.11	0.095	<0.001
	Size 20	-0.32 (0.09)	-0.50	-0.15	<0.001	.
	Size 18	Ref	.	.	.	.
Lesion Size in 4 levels	2.1-3 cm	0.28 (0.07)	0.14	0.43	<0.001	<0.001
	3.1-4 cm	0.39 (0.09)	0.21	0.58	<0.001	.
	>4 cm	0.32 (0.08)	0.17	0.48	<0.001	.
	N/A	0.14 (0.13)	-0.11	0.39	0.258	.
	0-2 cm	Ref	.	.	.	.
Lesion Score	3	0.28 (0.08)	0.12	0.45	0.001	0.003
	N/A	0.18 (0.09)	0.01	0.35	0.038	.
	2	Ref	.	.	.	.
Age (continuous)		0.01 (0.00)	0.00	0.01	0.002	0.002
Number of Lines of Chemo		0.05 (0.02)	0.01	0.09	0.008	0.008
BMI		-0.02 (0.01)	-0.03	-0.01	0.004	0.004
Time of Biopsy	1st Post-treatment	-0.02 (0.05)	-0.11	0.08	0.740	0.079
	2nd Post-treatment	-0.21 (0.09)	-0.39	-0.03	0.021	.
	3rd Post-treatment	-0.35 (0.26)	-0.85	0.16	0.176	.
	Baseline	Ref	.	.	.	.

Table 5b. Multivariable Analysis for Square Root Transform of Malignant Area for IR Biopsies

			95% CI			
**Factor**	**Levels**	**Estimate(Std)**	**Lower**	**Upper**	**Individual p Value**	**Overall p Value**
Age		0.01 (0.00)	0.00	0.01	<0.001	<0.001
BMI		-0.01 (0.01)	-0.02	-0.00	0.021	0.021
Needle Gauge	Size 14/16	0.45 (0.29)	-0.12	1.02	0.119	0.034
	Size 20	-0.24 (0.12)	-0.48	-0.01	0.042	
	Size 18	Ref	.	.	.	
Lesion Size	2.1-3 cm	0.26 (0.07)	0.13	0.40	<0.001	<0.001
	3.1-4 cm	0.38 (0.09)	0.21	0.56	<0.001	
	>4 cm	0.32 (0.08)	0.17	0.46	<0.001	
	N/A	0.19 (0.12)	-0.05	0.43	0.126	
	0-2 cm	Ref	.	.	.	
Biopsy Location	Deep	-0.25 (0.09)	-0.42	-0.07	0.006	0.005
	Superficial	-0.24 (0.10)	-0.43	-0.04	0.018	
	Thoracic	-0.23 (0.14)	-0.50	0.05	0.110	
	Bone	-0.83 (0.27)	-1.37	-0.29	0.003	
	Solid Organ	Ref	.	.	.	
Tumor Pathology	Anal Cancer	-0.05 (0.20)	-0.45	0.35	0.807	<0.001
	Appendiceal Tumor	-1.21 (0.20)	-1.60	-0.82	<0.001	
	Bone & Soft Tissue Tumor	-0.15 (0.18)	-0.50	0.20	0.389	
	Cervical Cancer	-0.18 (0.24)	-0.65	0.29	0.455	
	Colorectal Cancer	-0.94 (0.18)	-1.30	-0.58	<0.001	
	Endometrial Cancer	-0.30 (0.26)	-0.82	0.22	0.256	
	Head & Neck Cancer	-0.47 (0.28)	-1.01	0.07	0.086	
	Hepatocellular Carcinoma	-0.41 (0.25)	-0.90	0.08	0.099	
	Neuroendocrine Tumor	0.04 (0.19)	-0.34	0.42	0.821	
	Ovarian Cancer	-0.45 (0.22)	-0.89	-0.02	0.041	
	Pancreaticobiliary Cancer	-0.47 (0.19)	-0.85	-0.09	0.016	
	Peritoneal Cancer	-0.22 (0.22)	-0.66	0.22	0.325	
	Renal Cancer	-0.71 (0.22)	-1.14	-0.28	0.001	
	Skin Malignancy	-0.62 (0.22)	-1.05	-0.18	0.006	

			95% CI			
**Factor**	**Levels**	**Estimate(Std)**	**Lower**	**Upper**	**Individual p Value**	**Overall p Value**
	Small Bowel Cancer	-0.59 (0.22)	-1.03	-0.16	0.007	
	Thoracic Malignancy	-0.19 (0.23)	-0.64	0.25	0.394	
	Thyroid Malignancy	-0.49 (0.25)	-0.97	-0.00	0.049	
	Others	-0.23 (0.30)	-0.81	0.35	0.443	
	Breast Cancer	Ref	.	.	.	
Time of Biopsy	1st Post-treatment	-0.01 (0.05)	-0.10	0.08	0.871	0.027
	2nd Post-treatment	-0.25 (0.09)	-0.43	-0.07	0.006	
	3rd Post-treatment	-0.34 (0.25)	-0.83	0.15	0.176	
	Baseline	Ref	.	.	.	

Supplementary Table 6 Univariate and Multivariable Analysis of Factors Related to Presence of Inadequate Core(s) for IR Biopsies

Table 6a. Univariate Analysis of Factors Related to Presence of Inadequate Core(s)

Factor	Levels	Odds Ratio(95%CI)	Individual p-value	Overall p-value
Gender	Male	0.80 (0.60-1.06)	0.114	0.114
	Female	Reference	.	.
Biopsy of Primary	Yes	0.51 (0.30-0.88)	0.016	0.016
	No	Reference	.	.
Previous RT	Yes	1.30 (0.56-2.98)	0.543	0.543
	No	Reference	.	.
Disease Status	SD	1.00 (0.66-1.50)	0.990	0.617
	PR	1.42 (0.69-2.92)	0.335	.
	Untreated	0.74 (0.38-1.46)	0.386	.
	PD	Reference	.	.
Tumor Pathology	Anal Cancer	0.70 (0.28-1.79)	0.459	<0.001
	Appendiceal Tumor	12.22 (3.72-40.17)	<0.001	.
	Bone & Soft Tissue Tumor	0.72 (0.32-1.63)	0.434	.
	Cervical Cancer	0.88 (0.31-2.49)	0.803	.
	Colorectal Cancer	2.91 (1.21-6.98)	0.017	.
	Endometrial Cancer	0.92 (0.29-2.95)	0.890	.
	Head & Neck Cancer	1.13 (0.32-3.97)	0.853	.
	Hepatocellular Carcinoma	1.07 (0.35-3.30)	0.901	.
	Neuroendocrine Tumor	0.44 (0.18-1.07)	0.070	.
	Ovarian Cancer	1.30 (0.48-3.51)	0.604	.
	Pancreaticobiliary Cancer	1.28 (0.52-3.13)	0.590	.
	Peritoneal Malignancy	0.71 (0.26-1.94)	0.507	.
	Renal Cancer	1.01 (0.37-2.79)	0.984	.
	Skin Malignancy	1.52 (0.54-4.26)	0.428	.
	Small Bowel Cancer	1.82 (0.65-5.14)	0.256	.
	Thoracic Malignancy	0.66 (0.24-1.82)	0.420	.
	Thyroid Malignancy	1.84 (0.58-5.79)	0.300	.
	Others	1.42 (0.35-5.82)	0.625	.
	Breast Cancer	Reference	.	.

Factor	Levels	Odds Ratio(95%CI)	Individual p-value	Overall p-value
Experienced Proceduralist	Yes	1.05 (0.80-1.40)	0.709	0.709
	No	Reference	.	.
Presence of Assistant	Yes	1.02 (0.76-1.36)	0.903	0.903
	No	Reference	.	.
Needle Gauge	Size 18	0.80 (0.17-3.70)	0.770	0.250
	Size 20	1.14 (0.23-5.52)	0.875	.
	Size 14/16	Reference	.	.
Lesion Size 4-levels	2.1-3 cm	0.66 (0.46-0.95)	0.026	0.008
	3.1-4 cm	0.73 (0.46-1.16)	0.178	.
	>4 cm	0.51 (0.35-0.74)	<0.001	.
	N/A	0.93 (0.48-1.79)	0.831	.
	0-2 cm	Reference	.	.
Lesion Score	3	0.54 (0.35-0.82)	0.004	0.013
	N/A	0.66 (0.42-1.04)	0.072	.
	2	Reference	.	.
Age		0.98 (0.97-0.99)	0.002	0.002
Previous Lines of Chemotherapy		0.98 (0.91-1.05)	0.534	0.534
BMI		1.01 (0.98-1.03)	0.567	0.567
Time of Biopsy	1st Post-treatment	1.02 (0.78-1.35)	0.863	0.606
	2nd Post-treatment	1.44 (0.84-2.46)	0.182	.
	3rd Post-treatment	1.21 (0.31-4.71)	0.785	.
	Baseline	Reference	.	.

Table 6b. Multivariable Analysis of Factors Related to Presence of Inadequate Core(s) with Pathology

**Factor**	**Levels**	**Odds Ratio(95%CI)**	**Individual p Value**	**Overall p Value**
Age		0.98 (0.97-0.99)	<0.001	<0.001
Lesion size	2.1-3 cm	0.65 (0.45-0.96)	0.029	0.006
	3.1-4 cm	0.74 (0.45-1.20)	0.219	
	>4 cm	0.47 (0.31-0.70)	<0.001	
	N/A	0.84 (0.42-1.72)	0.641	
	0-2 cm	Reference		
Tumor Pathology	Anal Cancer	0.88 (0.34-2.27)	0.787	<0.001
	Appendiceal Tumor	16.96 (5.06-56.79)	<0.001	
	Bone & Soft Tissue	0.85 (0.37-1.94)	0.699	
	Cervical Cancer	0.81 (0.28-2.33)	0.690	
	Colorectal Cancer	3.38 (1.39-8.19)	0.007	
	Endometrial Cancer	1.30 (0.40-4.23)	0.660	
	Head & Neck Cancer	1.61 (0.45-5.81)	0.468	
	Hepatocellular Carcinoma	1.63 (0.51-5.17)	0.407	
	Neuroendocrine Tumor	0.54 (0.22-1.32)	0.173	
	Ovarian Cancer	1.51 (0.55-4.15)	0.422	
	Pancreaticobiliary Cancer	1.69 (0.68-4.23)	0.260	
	Peritoneal Malignancy	0.88 (0.31-2.48)	0.812	
	Renal Cancer	1.52 (0.54-4.32)	0.428	
	Skin Malignancy	2.10 (0.73-6.04)	0.166	
	Small Bowel Cancer	2.29 (0.80-6.55)	0.123	
	Thoracic Malignancy	1.05 (0.36-3.02)	0.933	
	Thyroid Malignancy	2.43 (0.75-7.86)	0.139	
	Others	1.92 (0.46-8.02)	0.373	
	Breast Cancer	Reference		
Time of Biopsy	1st Post-treatment	1.07 (0.80-1.44)	0.652	0.365
	2nd Post-treatment	1.67 (0.95-2.95)	0.077	
	3rd Post-treatment	1.26 (0.30-5.34)	0.754	
	Baseline	Reference		

Supplementary Table 7. Univariate and Multivariable Analysis of Factors Related to Zero Adequate Core(s) for IR Biopsies

Table 7a. Univariate Analysis of Factors Related to Zero Adequate Core(s)

Factor	Levels	Odds Ratio(95%CI)	Individual p-value	Overall p-value
Gender	Male	1.18 (0.81-1.72)	0.380	0.380
	Female	Reference	.	.
Biopsy of Primary	Yes	0.78 (0.36-1.67)	0.520	0.520
	No	Reference	.	.
Previous RT	Yes	0.85 (0.28-2.59)	0.777	0.777
	No	Reference	.	.
Tumor status	SD	1.68 (1.00-2.82)	0.051	0.088
	PR	2.10 (0.92-4.82)	0.080	.
	Untreated	0.61 (0.22-1.71)	0.349	.
	PD	Reference	.	.
Tumor Pathology	Anal Cancer	1.10 (0.17-7.13)	0.918	<0.001
	Appendiceal Tumor	25.44 (5.06-127.77)	<0.001	.
	Bone & Soft Tissue Tumor	2.10 (0.42-10.44)	0.363	.
	Cervical Cancer	2.24 (0.35-14.30)	0.393	.
	Colorectal Cancer	6.87 (1.41-33.54)	0.017	.
	Endometrial Cancer	1.95 (0.25-15.12)	0.522	.
	Head & Neck Cancer	0.93 (0.07-12.61)	0.956	.
	Hepatocellular Carcinoma	1.32 (0.15-11.47)	0.804	.
	Neuroendocrine Tumor	1.58 (0.29-8.71)	0.600	.
	Ovarian Cancer	2.18 (0.36-13.09)	0.393	.
	Pancreaticobiliary Cancer	1.65 (0.29-9.21)	0.570	.
	Peritoneal Malignancy	3.72 (0.64-21.61)	0.144	.
	Renal Cancer	5.99 (1.06-33.77)	0.042	.
	Skin Malignancy	5.76 (1.02-32.58)	0.048	.
	Small Bowel Cancer	4.83 (0.84-27.64)	0.077	.
	Thoracic Malignancy	3.04 (0.50-18.47)	0.226	.
	Thyroid Malignancy	5.68 (0.92-35.26)	0.062	.
	Others	8.26 (1.09-62.57)	0.041	.
	Breast Cancer	Reference	.	.

Factor	Levels	Odds Ratio(95%CI)	Individual p-value	Overall p-value
Biopsy Location 5-levels	Deep	1.59 (1.00-2.51)	0.048	0.038
	Superficial	1.51 (0.89-2.58)	0.127	.
	Thoracic	0.90 (0.48-1.67)	0.733	.
	Bone	5.30 (1.26-22.28)	0.023	.
	Solid Organ	Reference	.	.
Experienced Proceduralist	Yes	1.01 (0.70-1.46)	0.938	0.938
	No	Reference	.	.
Presence of Assistant	Yes	0.89 (0.60-1.30)	0.533	0.533
	No	Reference	.	.
Needle Gauge	Size 18	0.33 (0.06-1.72)	0.186	0.412
	Size 20	0.32 (0.06-1.79)	0.193	.
	Size 14/16	Reference	.	.
Lesion Size 4-levels	2.1-3 cm	0.61 (0.38-1.00)	0.051	0.029
	3.1-4 cm	0.54 (0.28-1.05)	0.069	.
	>4 cm	0.78 (0.49-1.26)	0.316	.
	Unavailable	1.78 (0.86-3.70)	0.122	.
	0-2 cm	Reference	.	.
Lesion Score	3	0.55 (0.33-0.90)	0.018	0.026
	Unavailable	0.83 (0.49-1.40)	0.478	.
	2	Reference	.	.
Patient Age		0.99 (0.97-1.00)	0.069	0.069
Previous Lines of Chemotherapy		0.85 (0.76-0.95)	0.004	0.004
BMI		1.04 (1.01-1.07)	0.012	0.012
Lesion Size		0.93 (0.86-1.01)	0.096	0.096
Time of Biopsy	1st Post-treatment	1.04 (0.73-1.49)	0.818	0.690
	2nd Post-treatment	1.47 (0.78-2.78)	0.230	.
	3rd Post-treatment	1.19 (0.22-6.59)	0.839	.
	Baseline	Reference	.	.

Table 7b. Multivariable Analysis of Factors Related to Presence of Zero Adequate Core(s) with Pathology Group

**Factor**	**Levels**	**Odds Ratio(95%CI)**	**Individual p Value**	**Overall p Value**
Age		0.98 (0.97-1.00)	0.037	0.037
Biopsy Location	Deep	1.28 (0.71-2.31)	0.411	0.040
	Superficial	1.79 (0.92-3.49)	0.088	
	Thoracic	0.97 (0.45-2.08)	0.931	
	Bone	9.66 (1.92-48.66)	0.006	
	Solid Organ	Reference		
Tumor Pathology	Anal Cancer	1.34 (0.20-8.96)	0.760	<0.001
	Appendiceal Tumor	27.76 (5.25-146.66)	<0.001	
	Bone & Soft Tissue	1.64 (0.32-8.42)	0.556	
	Cervical Cancer	2.08 (0.31-14.03)	0.451	
	Colorectal Cancer	8.68 (1.73-43.68)	0.009	
	Endometrial Cancer	1.62 (0.17-15.18)	0.670	
	Head & Neck Cancer	1.05 (0.07-14.83)	0.971	
	Hepatocellular Cancer	1.66 (0.18-15.51)	0.657	
	Neuroendocrine Tumor	1.82 (0.31-10.62)	0.505	
	Ovarian Cancer	2.27 (0.36-14.47)	0.384	
	Pancreaticobiliary Cancer	2.10 (0.36-12.16)	0.409	
	Peritoneal Malignancy	3.34 (0.54-20.51)	0.193	
	Renal Cancer	7.91 (1.30-48.06)	0.025	
	Skin Malignancy	6.16 (1.04-36.57)	0.046	
	Small Bowel Cancer	5.44 (0.91-32.39)	0.063	
	Thoracic Malignancy	3.79 (0.59-24.53)	0.161	
	Thyroid Malignancy	7.03 (1.01-48.96)	0.049	
	Others	5.67 (0.68-47.29)	0.109	
	Breast Cancer	Reference		
Disease Status	SD	1.79 (1.01-3.20)	0.047	0.046
	PR	2.29 (0.94-5.58)	0.068	
	New Disease	0.45 (0.14-1.46)	0.185	
	PD	Reference		
Time of Biopsy	1st Post-treatment	0.74 (0.46-1.19)	0.214	0.139
	2nd Post-treatment	1.78 (0.86-3.65)	0.119	

Factor	Levels	Odds Ratio(95%CI)	Individual p Value	Overall p Value
	3rd Post-treatment	0.99 (0.16-6.24)	0.994	
	Baseline	Reference		
